# Crisis management and the dilemma of rationing strategies in healthcare organizations

**DOI:** 10.1590/2175-8239-JBN-2018-0135

**Published:** 2018-10-18

**Authors:** José A. Moura-Neto, Ana Flavia Moura, José A. Moura

**Affiliations:** 1 Grupo CSB SalvadorBA Brasil Grupo CSB, Salvador, BA, Brasil.; 2 Escola Bahiana de Medicina e Saúde Pública SalvadorBA Brasil Escola Bahiana de Medicina e Saúde Pública, Salvador, BA, Brasil.

In May 2018, a severe supply crisis significantly impacted the healthcare system in Brazil. Due to popular dissatisfaction, coupled with the rising prices of diesel, a truck drivers' strike occurred in 24 Brazilian states. More than 500 roadblocks affected the supply system nationally^1^.

Railways are rare in Brazil and the country's internal transportation and supply system is strongly dependent on highways and the road network. Thus, the continental dimensions of Brazil makes it even more difficult to supply the interior regions of the country. Because of that, a concern emerged among the population: how many days could the country withstand having its entire supply system affected? Despite efforts by the Federal Government, delivery of healthcare supplies was interrupted in most regions, and many organizations adopted rationing strategies to avoid a complete lack of material.

Rationing strategies in health organizations is a controversial issue. Defining whether, when, how, and how much is not a trivial matter. Hence, this medical and managerial dilemma may lead to paralysis and inertia of decision makers. Further discussion and previous preparation for these moments may be the solution for better crisis management.

In hemodialysis facilities, the Brazilian supply crisis reached greater proportions since the lack of material would directly cause the death of patients in the short term. There are 758 hemodialysis centers in Brazil, directly responsible for 126,583 chronic patients in maintenance hemodialysis^2^. According to available data, when dialysis is discontinued, patients tend to live for six to eight days^3^^-^5. To avoid this scenario, rationing strategies were widely adopted by hemodialysis facilities. The challenge was to design strategies that could save constrained resources and not put patients' lives at elevated risk. To balance this and not cross the threshold of "acceptable risk", not only medical and technical knowledge were necessary, but also a dose of creativity.

Reduction of the dialysate flow and reduction of treatment time from a four-hour conventional session to a three-hour session were the most widely adopted strategies by the hemodialysis facilities. Hyperkalemia and hypervolemia were the main short-term medical concerns. Although less common, centers with a broader deficiency of health materials, such as needles and heparin, opted to alter the frequency of treatment - from three to two times a week.

Advocating for the reduction of hemodialysis doses in chronic kidney patients is an unusual situation for nephrologists. Although this decision was supported by most patients, it was a medical dilemma for many colleagues. In addition to deciding whether to ration, timing was also a challenge, perhaps a more difficult one.

We also adopted rationing strategies for one week in our hemodialysis facilities, implementing them since the interruption of deliveries until the reestablishment of normal supply. The reduction of the dialysate flow (500 to 300 mL per minute) and the reduction of the treatment time (4 to 3 hours per session) were preferred to the reduction of treatment frequency. Assistant physicians were instructed to individualize the medical evaluation and the decision for each patient to avoid hypervolemia. Therefore, patients with high interdialytic weight gain performed longer hemodialysis to ultrafiltrate and correct the fluid overload. Among around 1,700 hemodialysis patients in six hemodialysis facilities, increases in hospitalization and death rates were not reported.

In the whirlwind of the crisis, we also conducted an online survey among Brazilian nephrologists. A total of 50 nephrologists responded with their opinions regarding rationing strategies. The mean age of respondents was 43.8 years (SD = 10.90), 44% were medical directors or coordinators of dialysis facilities, and the majority (62%) were male.

Half of the respondents started rationing strategies immediately after supply discontinuation, while 40% adopted rationing strategies only when the internal inventory levels of the hemodialysis facility were considered low. No one disagreed about the need to adopt rationing strategies in the context of a severe supply crisis affecting the healthcare system.

Among the strategies adopted at the beginning of the rationing, the reduction of the dialysate flow (chosen by 68% of respondents) and the reduction of the treatment time (chosen by 52%) were the most common. The reduction of hemodialysis frequency from thrice- to twice-weekly was avoided and considered the last resource by 84% of nephrologists.

Difference between genders was also observed in this study. Additional regression analyses revealed that female respondents adopted significantly fewer simultaneous rationing strategies than male respondents (b = -0.44, SE = 0.12, *p* < 0.01). This result was robust after controlling for respondent age and function.

We believe our results are representative and in accordance with what was done in most hemodialysis facilities during this severe supply crisis. The strike lasted around ten days, but its repercussions extended for a few days after that until a complete supply normalization. Besides financial losses and political instability, the crisis taught us some lessons in crisis management. Finally, we hope that our experience and findings may help colleagues when dealing with the dilemma of rationing strategies in future crisis management situations.
